# Rheb promotes brown fat thermogenesis by Notch-dependent activation of the PKA signaling pathway

**DOI:** 10.1093/jmcb/mjz056

**Published:** 2019-07-22

**Authors:** Wen Meng, Xiuci Liang, Ting Xiao, Jing Wang, Jie Wen, Hairong Luo, Jianhui Teng, Yanquan Fei, Qinghai Zhang, Bilian Liu, Fang Hu, Juli Bai, Meilian Liu, Zhiguang Zhou, Feng Liu

**Affiliations:** 1 Department of Metabolism and Endocrinology, Second Xiangya Hospital, Central South University, Changsha 410011, China; 2 Metabolic Syndrome Research Center, Key Laboratory of Diabetes Immunology, Ministry of Education, National Clinical Research Center for Metabolic Diseases, Second Xiangya Hospital, Central South University, Changsha 410011, China; 3 Department of Pharmacology, University of Texas Health Science Center at San Antonio, San Antonio, TX, USA; 4 Department of Biochemistry and Molecular Biology, University of New Mexico Health Sciences Center, Albuquerque, NM, USA

**Keywords:** obesity, thermogenesis, beiging, adipose tissue, energy homeostasis

## Abstract

Increasing brown and beige fat thermogenesis have an anti-obesity effect and thus great metabolic benefits. However, the molecular mechanisms regulating brown and beige fat thermogenesis remain to be further elucidated. We recently found that fat-specific knockout of Rheb promoted beige fat thermogenesis. In the current study, we show that Rheb has distinct effects on thermogenic gene expression in brown and beige fat. Fat-specific knockout of Rheb decreased protein kinase A (PKA) activity and thermogenic gene expression in brown adipose tissue of high-fat diet-fed mice. On the other hand, overexpression of Rheb activated PKA and increased uncoupling protein 1 expression in brown adipocytes. Mechanistically, Rheb overexpression in brown adipocytes increased Notch expression, leading to disassociation of the regulatory subunit from the catalytic subunit of PKA and subsequent PKA activation. Our study demonstrates that Rheb, by selectively modulating thermogenic gene expression in brown and beige adipose tissues, plays an important role in regulating energy homeostasis.

## Introduction

Adipose tissue plays a central role in energy homeostasis via its profound effects on energy storage in the form of triglyceride lipids and energy dissipation by producing heat (thermogenesis) (Cinti, 2009; Harms and Seale, 2013; Cai et al., 2016). The energy storage function of adipose tissue is primarily achieved by white adipose tissue (WAT), while the thermogenic function of adipose tissue is accomplished by brown adipose tissue (BAT) and beige adipocytes (Harms and Seale, 2013; Lo and Sun, 2013; Nedergaard and Cannon, 2014; Hu et al., 2015; Lin et al., 2017; Luo et al., 2017; He et al., 2018). Excessive expansion of WAT due to positive energy balance and dysfunction in thermogenesis in BAT and beige fat are associated with obesity and various metabolic diseases.

We recently demonstrated that Ras homolog enriched in brain is a GTP-binding protein (Rheb), an upstream activator of the mechanistic target of rapamycin (mTOR), serves as a key negative regulator of beige fat thermogenesis, and plays a role in high-fat diet (HFD)-induced insulin resistance (Meng et al., 2017). However, while fat-specific knockout of Rheb greatly promotes protein kinase A (PKA) signaling and induces uncoupling protein 1 (UCP1) expression in subcutaneous WAT, Rheb deficiency had no significant effect on PKA signaling and UCP1 expression in BAT under normal diet (ND) feeding conditions (Meng et al., 2017), suggesting that Rheb has distinct effects on PKA activity and thermogenic programing in WAT and BAT.

The mechanism by which Rheb differentially regulates thermogenic gene expression in WAT and BAT is unclear. We previously found that rapamycin treatment only partially alleviated the suppressing effect of Rheb on UCP1 expression in primary white adipocytes, suggesting that Rheb suppresses beige fat thermogenesis via an mTOR complex 1 (mTORC1)-independent mechanism (Meng et al., 2017). In addition to mTORC1, Rheb has been shown to regulate the Notch signaling pathway in the Drosophila external sensory organ and certain mammalian cells (Karbowniczek et al., 2010; Heard et al., 2014). The Notch is a cell membrane protein and is activated by ligand binding-induced and γ-secretase–mediated proteolytic cleavage. The released intracellular domain of Notch (NICD) subsequently enters the nucleus of the cells to modify gene expression to regulate a variety of important biological events such as inducing neurons to die or to survive and suppressing oncogenic and tumor cell growth (Bray, 2016). However, the roles of Notch signaling in adipocytes remain obscure. Cell culture studies show that Notch either inhibits, promotes, or is dispensable for adipogenesis (deCelis and Bray, 1997; Fan et al., 2004; Becam et al., 2010; Sprinzak et al., 2010; Liu et al., 2015). Nevertheless, *in vivo* studies showed that adipocyte-specific ablation of Notch promoted beige fat development, accompanied by improved glucose tolerance and insulin sensitivity (Bi et al., 2014). However, the mechanisms underlying the functional role of Notch signaling in regulating thermogenic gene expression in BAT are poorly understood.

In the current study, we show that fat-specific disruption of Rheb expression inhibits PKA activity and thermogenic gene expression in BAT of HFD-fed mice. In addition, we demonstrate that Rheb promotes PKA activity and UCP1 expression in brown adipocytes via activating the Notch signaling pathway. Mechanistically, Notch signaling activates PKA by promoting the dissociation of the regulatory subunit (RIIβ) from the catalytic subunit (C) of PKA in brown adipocytes. Taken together, these studies demonstrate that Notch signaling plays opposite roles in regulating thermogenesis in WAT and BAT. In addition, Rheb selectively activates PKA signaling via Notch in brown adipocytes and promotes thermogenic gene expression in BAT.

## Results

### Fat-specific knockout of Rheb suppresses PKA pathway and UCP1 expression in BAT

**Figure 1:**
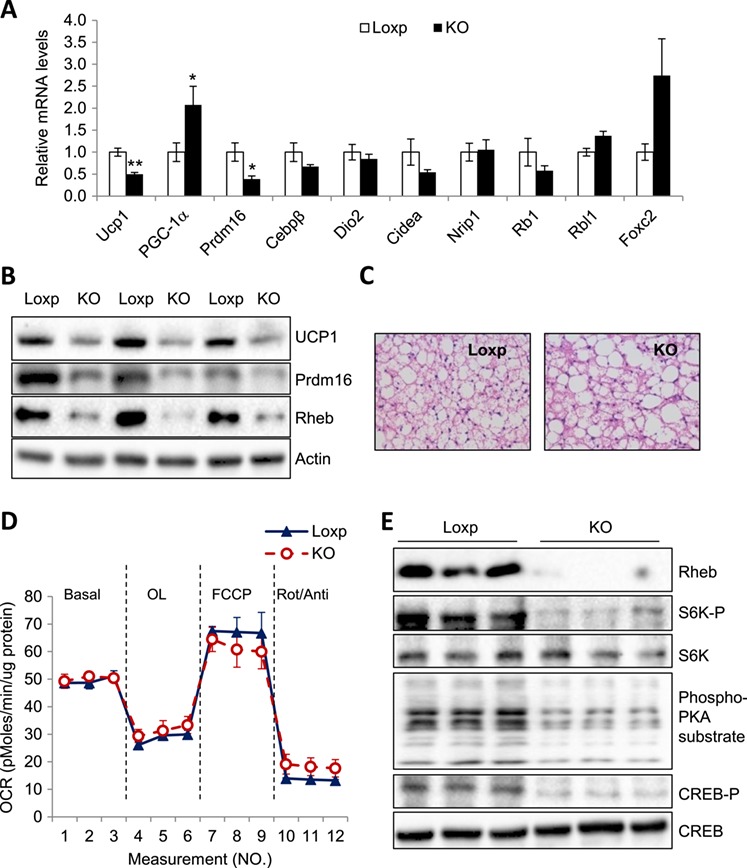
UCP1 expression and PKA activity are suppressed by Rheb deficiency in BAT under HFD-feeding condition**.** Eight-week-old Rheb^fKO^ (KO) and Loxp control mice were fed with HFD for 17 weeks. (**A**) Quantitative real-time PCR analysis of thermogenic genes in BAT from KO and Loxp control mice (*n* = 6/groups). All genes were normalized to actin. Data are presented as mean ± SEM. ^⋆^*P* < 0.05; ^⋆⋆^*P* < 0.01. (**B**) Western blot analysis of the protein levels of UCP1 and Prdm16 in BAT of KO and Loxp control mice. (**C**) Representative images of hematoxylin and eosin staining of BAT sections from HFD-fed KO and Loxp control mice. (**D**) OCR of primary brown adipocytes of KO and Loxp control mice. VO_2_ consumption was normalized to protein content. OL, oligomycin; Rot, rotenone; Anti, antimycin. (**E**) Western blot analysis of the protein levels of CREB-P and phosphorylation of PKA substrate in BAT of KO and Loxp control mice.

Our recent study reveals that Rheb is a key regulator of thermogenesis in adipose tissue (Meng et al., 2017). However, while Rheb deficiency in adipose tissue significantly promoted the expression levels of thermogenic genes in sWAT of HFD-fed Rheb^fKO^ mice (Meng et al., 2017), to our surprise, we found that messenger RNA (mRNA) levels of thermogenic genes such as *ucp1* and *prdm16* were significantly reduced in BAT of the HFD-fed Rheb^fKO^ mice compared to control mice despite an induction of peroxisome proliferator-activated receptor gamma coactivator 1-alpha (PGC-1α) (Figure 1A). Decreased protein levels of UCP1 and Prdm16 were also observed by western blot in BAT of the HFD-fed Rheb^fKO^ mice compared to control mice (Figure 1B). Furthermore, a greater number of larger lipid droplets are found in BAT of the HFD-fed Rheb^fKO^ mice than those in control littermates (Figure 1C). Nevertheless, there was no significant difference in oxygen consumption rate (OCR) in brown adipocytes between HFD-fed Rheb^fKO^ mice and wild-type littermates (Figure 1D), suggesting that Rheb deficiency had no significant effect on metabolic rate and/or substrate utilization in BAT of mice.

Because Rheb deficiency activated the PKA signaling pathway in sWAT (Meng et al., 2017), we asked whether Rheb promotes thermogenic gene expression by activating PKA in BAT. To this end, we examined the phosphorylation of CREB at Ser^133^, a direct PKA phosphorylation site (Cao et al., 2001), in BAT of HFD-fed Rheb^fKO^ and Loxp control mice. The phosphorylation of CREB at Ser^133^ and other PKA substrates was greatly reduced in BAT of the Rheb^fKO^ mice compared to Loxp control mice (Figure 1E). Taken together with the findings that the mRNA and protein levels of UCP1 were significantly decreased in BAT of the Rheb^fKO^ mice compared to Loxp control mice (Figure 1A and B), these results reveal that, unlike in beige fat, Rheb positively regulates PKA signaling and UCP1 expression in BAT.

### Rheb promotes UCP1 expression via mTORC1-independent signaling pathway in brown adipocytes

**Figure 2:**
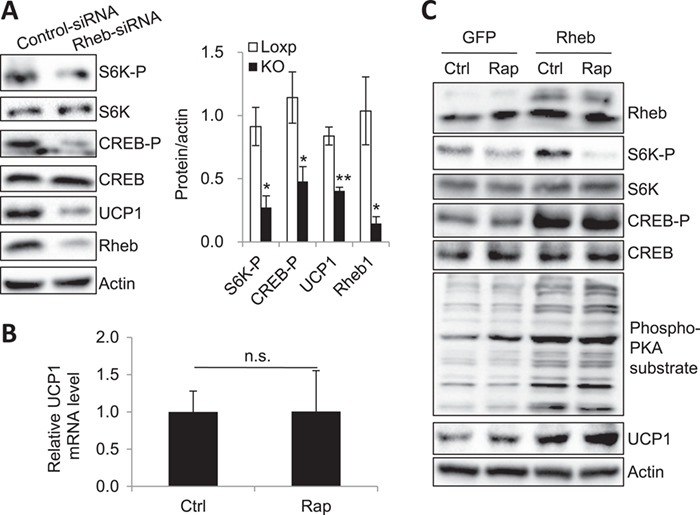
Rheb promotes UCP1 expression via a mTORC1-independent signaling pathway in brown adipocytes. (**A**) Primary brown adipocytes were treated with a Rheb-specific siRNA or a siRNA control for 48 h. Cells were lysed, and the expression of S6K-P, CREB-P, UCP1, and Rheb proteins was determined by western blot. Data were representative of three independent experiments each with a similar result. Data were presented as mean ± SEM. ^⋆^*P* < 0.05; ^⋆⋆^*P* < 0.01. (**B**) The mRNA level of UCP1 in primary brown adipocytes, which were treated with or without 20 nM rapamycin (Rap) for 24 h. Data were presented as mean ± SEM. (**C**) Primary brown adipocytes were infected with Lentiviruses encoding GFP or GFP plus Rheb and then were induced to differentiation. Cells were treated with or without 20 nM rapamycin (Rap) for 24 h and lysed. The phosphorylation and expression of proteins in cell lysates were determined by western blot. Data are representative of three independent experiments with a similar result.

To determine whether Rheb has a cell-autonomous role in regulating PKA signaling, we examined whether ablation of Rheb affects PKA signaling in primary adipocytes isolated from BAT of C57BL/6 mice. Suppressing Rheb expression by small interfering RNA (siRNA) greatly inhibited the phosphorylation of CREB and markedly decreased the expression of UCP1 in primary adipocytes isolated from BAT of C57BL/6 mice (Figure 2A). However, inhibition of mTORC1 by rapamycin had no significant effect on UCP1 mRNA expression in primary brown adipocytes (Figure 2B), suggesting that Rheb deficiency suppresses UCP1 via an mTORC1-independent mechanism in brown adipocytes. To further test this, we treated primary brown adipocytes overexpressing Rheb with or without rapamycin. Overexpression of Rheb promoted the phosphorylation of PKA substrates and CREB and increased UCP1 expression in brown adipocytes (Figure 2C). Treating the cells with rapamycin greatly inhibited S6K phosphorylation, but had no significant effect on the promoting role of Rheb in PKA substrates and CREB phosphorylation (Figure 2C), indicating that Rheb stimulates PKA signaling and UCP1 expression via an mTORC1-independent mechanism in brown adipocytes.

### Rheb stimulates the Notch signaling pathway in brown adipocytes but not in white adipocytes

**Figure 3:**
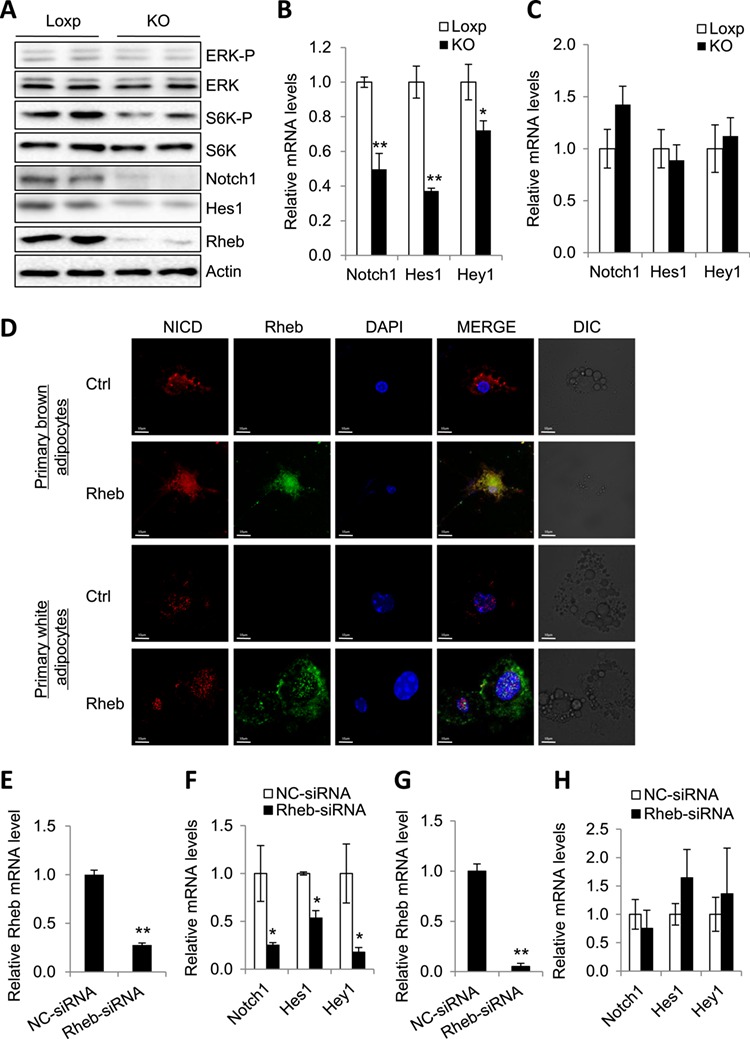
Rheb regulates the Notch signaling pathway in brown adipocytes. (**A**) ERK, Notch, and mTORC1 signaling in BAT of Rheb^fKO^ and control mice were examined by western blot. Data were representative of three independent experiments with a similar result. (**B** and **C**) The mRNA levels of genes in the Notch signaling pathway in BAT (**B**) and sWAT (**C**) from Rheb^fKO^ and control mice fed with an HFD (*n* = 6/groups). The expression levels for each gene were normalized to those of actin. Data are presented as mean ± SEM. ^⋆^*P* < 0.05; ^⋆⋆^*P* < 0.01. (**D**) Immunofluorescence analysis of NICD cellular localization in primary brown and white adipocytes of Rheb overexpression. Cells were immunofluorescence stained with the released NICD and Rheb antibodies. (**E**–**H**) Primary brown adipocytes (**E** and **F**) and primary white adipocytes (**G** and **H**) were treated with a Rheb-specific siRNA or a siRNA control for 48 h followed by harvest and quantitative real-time PCR. Data were presented as mean ± SEM. ^⋆^*P* < 0.05; ^⋆⋆^*P* < 0.01.

Rheb has been shown to regulate three major signaling pathways, including the mTORC1 signaling pathway, the B-Raf signaling pathway, and the Notch signaling pathway in mammalian cells (Karbowniczek et al., 2010; Heard et al., 2014). Given that fat-specific knockout of Rheb significantly inhibited the phosphorylation of S6K but not the ERK in BAT (Figure 3A), it is unlikely that alteration in the B-Raf signaling pathway plays a major role in the down-regulation of thermogenic gene expression in BAT of the Rheb^fKO^ mice. To determine whether the Notch signaling pathway plays a role in Rheb-induced UCP1 expression in BAT of the Rheb^fKO^ mice, we examined the expression of key components in the Notch signaling pathway in BAT of Rheb^fKO^ and Loxp control mice. Depletion of Rheb significantly suppressed the protein and mRNA levels of key components in the Notch signaling pathway, including Notch1 and Hes1, in BAT of the mice (Figure 3B), but not in sWAT of mice (Figure 3C). In addition, we found that overexpression of Rheb in primary brown adipocytes promoted nuclear translocation of NICD from the cytosol (Figure 3D). On the other hand, NICD was largely localized in the nucleus of primary white adipocytes, and Rheb overexpression had no significant effect on NICD localization in primary white adipocytes (Figure 3D), suggesting the presence of a mechanism by which Rheb selectively activates the Notch signaling pathway in brown but not in white adipocytes. To determine whether Rheb regulates Notch signaling in a cell-autonomous manner, we examined Notch signaling in brown adipocytes in which the expression of Rheb is suppressed by siRNA (Figure 3E). Knockdown of Rheb significantly suppressed the mRNA levels of key components in the Notch signaling pathway in primary brown adipocytes (Figure 3F), indicating that Rheb positively regulates Notch signaling pathway in a cell-autonomous manner. However, suppressing Rheb expression had no significant effect on Notch signaling in primary white adipocytes (Figure 3G and H), further demonstrating a cell-specific role of Rheb in regulating Notch signaling in brown and white adipocytes.

### Rheb increases PKA signaling and UCP1 expression via the Notch signaling pathway in brown adipocytes

**Figure 4:**
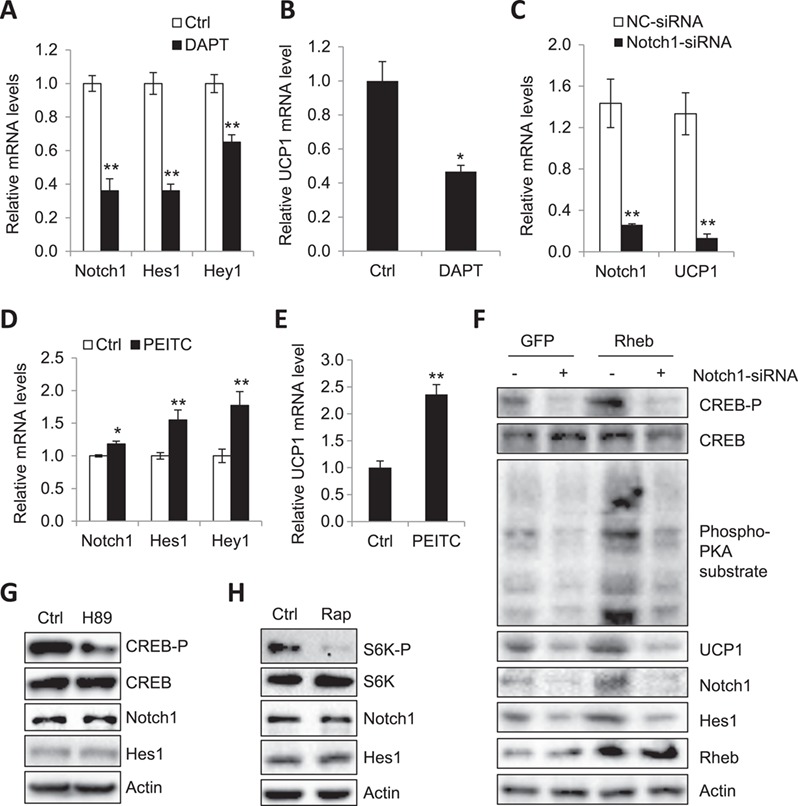
Rheb via Notch promotes PKA-dependent UCP1 expression in brown adipocytes. (**A** and **B**) Quantitative real-time PCR analysis of the Notch signaling target genes (**A**) and UCP1 mRNA level (**B**) in primary brown adipocytes, which were treated with or without 10 μM DAPT for 24 h. Data were mean ± SEM. ^⋆^*P* < 0.05; ^⋆⋆^*P* < 0.01. (**C**) Primary brown adipocytes were treated with a Notch1-specific siRNA or a siRNA control for 48 h and followed by harvest and quantitative real-time PCR. (**D** and **E**) Quantitative real-time PCR analysis of the Notch signaling target genes (**D**) and UCP1 mRNA level (**E**) in primary brown adipocytes treated with or without 5 μM PEITC for 24 h. Data were presented as mean ± SEM. ^⋆^*P* < 0.05; ^⋆⋆^*P* < 0.01. (**F**) Primary brown adipocytes were infected with Lentivirus encoding GFP or GFP plus Rheb and then were induced to differentiation. Cells were treated with a Notch1-specific siRNA or a siRNA control for 48 h and analyzed for protein expression using the indicated antibodies. Data were representative of three independent experiments each with a similar result. (**G**) Primary brown adipocytes were isolated and induced to differentiation. Cells were treated with or without 10 nM H89 for 24 h followed by harvest and WB. Data were representative of three independent experiments each with a similar result. (**H**) Western blot analyses of the Notch1 and Hes1 protein level in primary brown adipocytes, which were treated with or without 20 nM rapamycin for 24 h. Data were representative of three independent experiments each with a similar result.

To determine whether activation of the Notch signaling pathway mediates UCP1 expression, we treated primary brown adipocytes with DAPT, a γ-secretase inhibitor that indirectly inhibits Notch (Bi et al., 2014). DAPT treatment significantly suppressed the mRNA levels of Hes1, Hey1, and Notch1 in primary brown adipocytes (Figure 4A), demonstrating the efficiency of the inhibitor. Treating brown adipocytes with DAPT significantly reduced the mRNA levels of UCP1 (Figure 4B), suggesting a promoting effect of the Notch signaling pathway on thermogenic gene expression. In agreement with this, suppressing Notch expression by siRNA significantly reduced the expression levels of UCP1 in primary brown adipocytes (Figure 4C). In addition, treating primary brown adipocyte with phenethyl isothiocyanate (PEITC), a Notch signaling pathway activator, greatly stimulated UCP1 expression in primary brown adipocytes (Figure 4D and E). To determine the potential mechanism by which Notch signaling pathway promotes UCP1 gene expression in brown adipocytes, we examined the phosphorylation of CREB and other PKA substrates in Rheb-overexpressed primary brown adipocytes. Rheb overexpression greatly increased the phosphorylation of CREB and PKA substrates (Figure 4F; Supplementary Figure S1A). The stimulatory effect of Rheb on PKA activation was markedly suppressed by treating cells with Notch1-siRNA or DAPT (Figure 4F; Supplementary Figure S1A). Consistent with these results, Notch1-siRNA or DAPT treatment markedly blocked Rheb-induced UCP1 expression in brown adipocytes (Figure 4F; Supplementary Figure S1A), suggesting the Rheb-increased thermogenic gene expression is mediated by the activation of Notch signaling. Importantly, inhibition of PKA activity by H89 (Figure 4G; Supplementary Figure S1B) or inactivation of mTORC1 by rapamycin (Figure 4H; Supplementary Figure S1C) did not suppress the mRNA and protein levels of Notch1 and Hes1, suggesting that Rheb regulates Notch signaling pathway via a PKA- and mTORC1-independent mechanism.

### Notch signaling activates PKA by suppressing the binding of the regulatory subunit to the catalytic subunit of PKA in brown adipocytes

**Figure 5:**
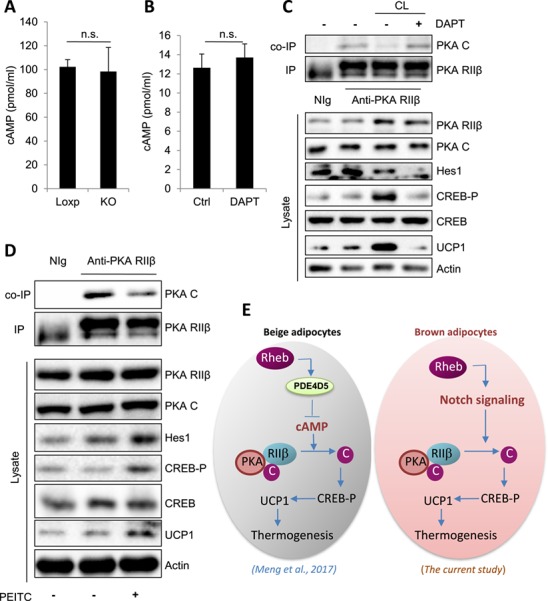
Notch signaling regulates PKA activation by interfering the binding of PKA RIIβ and PKA C subunits in brown adipocytes. (**A**) cAMP levels in BAT of Rheb^fKO^ (KO) and Loxp mice (*n* = 3/group). (**B**) cAMP levels in the primary brown adipocytes were treated with or without DAPT (10 μM) for 24 h. Data are presented as mean ± SEM. ^⋆^*P* < 0.05; ^⋆⋆^*P* < 0.01. (**C**) The immunoprecipitation (IP) of PKA RIIβ and coimmunoprecipitation (co-IP) of PKA C in CL 316,243 (CL) treated primary brown adipocytes, which were treated with or without DAPT (10 μM) for 24 h. (**D**) Primary brown adipocytes were treated with or without PEITC (5 μM) for 24 h. The interaction of PKA RIIβ and PKA C was determined by immunoprecipitation experiments. Data were representative of three independent experiments each with a similar result. (**E**) A proposed model on the distinct mechanisms by which Rheb regulates thermogenic gene expression in brown and beige adipocytes. In beige adipocytes, Rheb inhibits thermogenic genes expression by inhibiting the cAMP–PKA signaling pathway (Meng et al., 2017). On the other hand, Rheb promotes thermogenic genes expression via Notch-mediated activation of the PKA signaling pathway in brown adipocytes.

To determine the mechanism by which Notch signaling regulates PKA activation in brown adipocytes, we first examined cyclic adenosine monophosphate (cAMP) levels in BAT of Rheb^fKO^ mice. Rheb ablation in adipose tissue had no significant effect on cAMP levels in mouse BAT (Figure 5A). Consistent with this result, DAPT treatment did not affect intracellular cAMP levels (Figure 5B), suggesting that Notch signaling regulated PKA activation by acting at a site downstream of cAMP in the PKA signaling pathway in brown adipocytes.

CL 316,243, a β3-AR agonist, stimulates thermogenesis via increased cAMP levels that dissociate the catalytic subunit (PKA C) from the regulatory subunit (RIIβ), rendering PKA activation (Zhang et al., 2012). Treating brown adipocytes with the Notch inhibitor, DAPT suppressed CL 316,243 induced dissociation between the catalytic and regulatory subunit of PKA (Figure 5C). Consistent with these results, DAPT treatment markedly suppressed Rheb-induced dissociation between the catalytic and regulatory subunit of PKA in brown adipocytes (Supplementary Figure S2A). On the other hand, activation of Notch signaling by treating cells with PEITC promoted the dissociation between PKA C and the RIIβ subunits of PKA in primary brown adipocytes (Figure 5D). Together, these data suggest that Notch signaling activates PKA by inhibiting the binding of the regulatory subunit to the catalytic subunit of PKA in brown adipocytes (Figure 5E).

## Discussion

We recently found that Rheb negatively regulates beige fat thermogenesis and plays a potential role in diet-induced insulin resistance (Meng et al., 2017). In the current study, we show that fat-specific knockout of Rheb decreased PKA activity and thermogenesis function in BAT of HFD-fed mice (Figure 1A, B, and E), suggesting a positive role of Rheb in regulating BAT thermogenesis and the presence of distinct mechanisms by which Rheb regulates thermogenesis in white and brown adipocytes. In addition, we found that Rheb stimulates PKA signaling and UCP1 expression via an mTORC1-independent mechanism in brown adipocytes (Figure 2A–C). Furthermore, we demonstrate that fat-specific knockout of Rheb suppresses BAT thermogenesis by inhibiting the Notch–PKA signaling pathway in mouse BAT (Figure 4A–F). Our results reveal a previously unrecognized mechanism by which Rheb regulates thermogenic gene expression in brown adipocytes.

We found that the expression level of UCP1 was significantly reduced in BAT of the HFD-fed Rheb^fKO^ mice compared to control mice despite an induction of PGC-1α expression (Figure 1A). It is unclear why UCP1 expression was decreased while PGC-1α expression remained high in BAT of Rheb^fKO^ mice. However, a recent study showed that IRF4 was a key thermogenic transcriptional partner of PGC-1α, which was sufficient to promote increased thermogenic gene expression in BAT (Kong et al., 2014). Interestingly, we found that IRF4 was significantly decreased in BAT of the HFD-fed Rheb^fKO^ mice (Supplementary Figure S2B), suggesting a possible mechanism by which Rheb deficiency reduces UCP1 expression in BAT.

Our study shows that Rheb is required for Notch signaling activation in brown adipocytes but not in white adipocytes (Figure 3B, C, and E–H). In addition, we found that the activated form of Notch (NICD) is principally localized in the cytoplasm of brown adipocytes and co-localized with Rheb (Figure 3D). On the other hand, NICD is co-localized mainly in the nucleus of white adipocytes (Figure 3D). The distinct localization of NICD may provide a mechanism by which Rheb regulates Notch signaling in brown and white adipocytes.

We found that Rheb promotes UCP1 expression in BAT of HFD-fed mice but not in ND-fed mice (Figure 1A and B; Meng et al., 2017). These findings are interesting given that emerging evidence has implicated that excessive caloric intake enhances energy expenditure and thus limits weight gain in rodents (Bachman et al., 2002; Kazak et al., 2017; Okla et al., 2017; Zhang et al., 2018). In agreement with these results, UCP1 mRNA and protein expression levels were induced in BAT (Supplementary Figure S3A and C) but decreased in sWAT of HFD-fed mice (Supplementary Figure S3B and D), suggesting that Rheb may mediate HFD-induced thermogenic gene expression. Future studies will be needed to elucidate the precise mechanism by which HFD feeding synergizes the role of Rheb in regulating thermogenic gene expression in BAT.

The roles of Notch signaling in regulating adipocyte homeostasis remain to be further elucidated (Bi and Kuang, 2015). Inhibition of Notch signaling has been shown to promote beiging and thermogenesis in WAT (Bi et al., 2014). Consistent with this finding, we found that inhibition of Notch signaling by treating primary white adipocytes with DAPT activated PKA, as demonstrated by increased CREB phosphorylation and UCP1 expression (Supplementary Figure S4A and B). These results show that Notch signaling negatively regulates thermogenesis in WAT. However, inhibition of Notch signaling suppressed PKA activation and thermogenic gene expression in brown adipocytes (Figure 4A–C and F). In addition, activation of Notch signaling induced UCP1 expression in brown adipocytes (Figure 4D and E). An unanswered question is how Notch signaling exerts opposite effects on thermogenic gene expression in white and brown adipocytes. Nevertheless, we found that the NICD was preferentially localized in the cytoplasm of primary brown but the nucleus of primary white adipocytes (Figure 3D), suggesting a potential mechanism by which Rheb specifically regulates thermogenesis in white adipocytes and brown adipocytes.

In summary, we present evidence showing the presence of a distinct mechanism by which Rheb regulates thermogenesis in beige and brown adipocytes (Figure 5E). We demonstrate that fat-specific disruption of Rheb expression inhibits thermogenic gene expression via an mTORC1-independent mechanism in brown adipocytes. Lastly, we show that Rheb promotes PKA signaling and thermogenic gene expression in brown adipocytes via activation of the Notch signaling pathway, uncovering a novel mechanism regulating thermogenic gene expression in brown adipocytes. Distinct regulation of thermogenic gene expression by Rheb in beige and brown adipocytes may provide a mechanism by which energy homeostasis is fine-turning regulated *in vivo*.

## Materials and methods

### Antibodies

Antibodies against PKA RIIβ, activated Notch1 (NICD), Alexa Fluor® 568 (ab175470), and Alexa Fluor® 488 (ab150117) (1:400) were from Abcam. The anti-UCP1 antibody was from Sigma-Aldrich. The anti-flag antibody was from Kaijing Biotechnology Co. Ltd. All other antibodies were from Cell Signaling Technologies.

### Generation of fat tissue-specific KO mice

Adipose tissue-specific Rheb knockout mice (Rheb^fKO^) were described previously (Meng et al., 2017). The floxed littermates were used as controls. For the HFD-induced obesity experiments, the mice were randomly distributed into weight-matched groups fed either a normal chow diet (ND) or an HFD (60 kcal% fat, Catalog no. D12492; Research Diets Inc.). All animals were housed in a temperature-controlled environment with a 12 h:12 h light/dark cycle and had access to food and water *ad libitum*. All animal studies were performed under a protocol approved by the Central South University Animal Care and Use Committee.

### Primary cell culture

Primary stromal vascular fractions from either subcutaneous white or interscapular brown fat depots were isolated from 4-week-old C57BL/6J mice and cultured as described previously (Liu et al., 2014). Differentiation of adipocytes was performed according to the procedure as described previously (Fasshauer et al., 2001). Differentiated primary adipocytes were treated with a Rheb-specific siRNA or a siRNA control for 48 h, and followed by harvest and quantitative real-time polymerase chain reaction (PCR) or western blot.

### Immunofluorescence experiments

Primary brown and white adipocytes grown on coverslips were fixed with 4% paraformaldehyde and then permeabilized by Triton X-100 (0.1%) in phosphate-buffered saline (PBS) for 10 min. The cells were then blocked with 5% bovine serum albumin (BSA) in PBS for 30 min and stained with first antibodies (anti-NICD or Flag) overnight. After washing, the fixed cells were then stained with an anti-rabbit Alexa Fluor® 568 antibody (1:400) or an anti-mouse Alexa Fluor® 488 antibody (1:400) for 60 min. Cell nuclear DNAs were detected by DAPI Staining Solution (Sigma). Confocal images were taken by a Zeiss LSM 780 confocal microscope.

### OCR analysis

Mitochondrial OCR in intact cells was measured using Seahorse Bioscience XF-24 analyzer (Seahorse Bioscience) according to the procedure as described in the manufacturer's instruction. Briefly, primary preadipocytes were seeded into XF-24 microplates and induced to differentiation at 37°C with 5% CO_2_. Cells were maintained at 37°C in a non-CO_2_ incubator for at least 1 h before assays. Adenosine triphosphate turnover and maximal uncoupled OCRs were determined by treating the cells with oligomycin (1 μM) (Sigma-Aldrich) or FCCP (1 μM) (Sigma-Aldrich), respectively. Rotenone and antimycin A (1 μM each) (Sigma-Aldrich) were used to inhibit Complex 1- and Complex 3-dependent respiration. OCR was normalized to protein content. Each experimental condition was analyzed using four to six biological replicates (Kong et al., 2015).

### cAMP assay

cAMP levels in tissue or cells were determined using a colorimetric cAMP ELISA kit (Cell Biolabs), according to the procedure described in our previous study (Meng et al., 2017).

### Generation of lentiviruses encoding Rheb

Rheb lentiviruses and control viruses were generated by co-transfecting HEK293T cells with pWPI-Rheb or pWPI-control plasmid, respectively, together with packaging plasmid pMD2.G and envelope plasmid pAX2A. After 48 h, supernatant media containing viruses were collected and used to infect primary preadipocytes.

### Protein extraction and western blot analysis

For protein extraction, ~30 mg frozen tissue was homogenized in 400 μl radioimmunoprecipitation assay buffer (Beyotime Institute of Biotechnology) supplemented with complete protease inhibitor cocktail (Roche). Extracts were spun down and the fat layer and cell debris were removed. Protein concentration was determined by bicinchoninic acid assay (Thermo). An equal amount of proteins from each sample was loaded and separated by sodium dodecyl sulfate-polyacrylamide gel electrophoresis. Proteins were transferred to polyvinylidene difluoride membrane and incubated with a blocking buffer (5% BSA in 20 mM Tris-HCl, pH 7.5, 137 mM NaCl, and 0.1% Tween-20) for 1 h at room temperature and then incubated with primary antibodies at 4°C overnight. The membrane was incubated with secondary antibodies (1:5000 to 1:10000 dilution) for 1 h at room temperature and detected with enhanced chemiluminescence (Bio-Rad).

### Real-time PCR

Tissue samples were homogenized in Trizol (Invitrogen) and total RNA was isolated according to the manufacturer’s suggested protocol. One microgram of RNA was used for complementary DNA synthesis (Thermo). Quantitative PCR reactions were performed using the SYBR green mix (Roche) and quantitated using Applied Biosystems 7900 HT sequence detection system. Duplicate runs of each sample were normalized to β-actin to determine relative expression levels.

### Immunoprecipitation and western blot

For western blot analysis, differentiated primary brown adipocytes were treated with or without CL 316,243 (CL; Sigma-Aldrich) (10 nM), which follows DAPT (Sigma-Aldrich) (10 μM) treatment for 1 h, or PEITC (Sigma-Aldrich) (5μM) for 24 h, and then these cells lysed in immunoprecipitation assay buffer, and PKA RIIβ protein was immunoprecipitated using anti-PKA RIIβ antibody (ab75993; Abcam). Proteins coimmunoprecipitated with PKA C were determined using anti-PKA C antibody (4782; CST).

### Statistical analysis

Statistical analysis was performed using SPSS software version 19.0 (SPSS Inc.). Statistical analysis of the data was performed using analysis of variance or Student’s *t*-test. Data shown are average ± SEM. The *P* value of ≤0.05 was considered to be statistically significant.

## Supplementary Material

Supplementary_Materials-06-12-19-JMCB_mjz056
