# Supplementary material for: Rheb promotes brown fat thermogenesis by Notch-dependent activation of the PKA signaling pathway
Source: J Mol Cell Biol. 2019 Jul 22;11(9):781–90. doi: 10.1093/jmcb/mjz056 (PMC6821354; doi:10.1093/jmcb/mjz056)
Supplement: Supplementary_Materials-06-12-19-JMCB_mjz056 [file supplementary_materials-06-12-19-jmcb_mjz056.pdf]

Supplementary Material

Rheb Promotes Brown Fat Thermogenesis by Notch-dependent Activation of the PKA Signaling Pathway

Wen Meng [1,2,*], Xiuci Liang[1,2], Ting Xiao[1,2], Jing Wang[1,2], Jie Wen[1,2], Hairong Luo[1,2], Jianhui Teng[1,2], Yanquan Fei[1,2], Qinghai Zhang[1,2], Bilian Liu[1,2], Fang Hu[1,2], Juli Bai[1,2,3], Meilian Liu[1,2,4], Zhiguang Zhou[1,2], and Feng Liu[1,2,3*]

[1]Department of Metabolism and Endocrinology, Second Xiangya Hospital, Central South University, Changsha, Hunan 410011, China;

[2]Metabolic Syndrome Research Center, Key Laboratory of Diabetes Immunology, Ministry of Education, National Clinical Research Center for Metabolic Diseases, Second Xiangya Hospital, Central South University, Changsha, Hunan 410011, China;

[3]Department of Pharmacology, University of Texas Health Science Center at San Antonio, San Antonio, TX

[4]Department of Biochemistry and Molecular Biology, University of New Mexico Health Sciences Center, Albuquerque, NM

*Correspondence should be addressed to: Wen Meng (122501006@csu.edu.cn) or Feng Liu (liuf@uthscsa.edu)

Contents

I. Supplementary Figures……………………………………………………………………...3

Supplementary Figure S1. Rheb regulates PKA-UCP1 signaling pathway via Notch signaling pathway in brown adipocytes ………………………………………………………………….3

Supplementary Figure S2. Notch signaling activates PKA by suppressing the binding of the regulatory subunit to the catalytic subunit of PKA in brown adipocytes………………………...4

Supplementary Figure S3. HFD induced Ucp1 expression in BAT of mice, but inhibited in Swat……………………………………………………………………………………………5

Supplementary Figure S4. Inhibition of Notch signaling promotes browning of white adipose tissue…………………………………………………………………………………………...6

Supplementary Figure S5. Rheb deficiency could suppress cold-induced UCP1 level in BAT of HFD-fed $Rheb^{fKO}$……………………………………………………………………………...7

II. Supplementary table……………………………………………………………………......8

Supplementary Table S1. Antibody information..8

I. Supplementary Figures

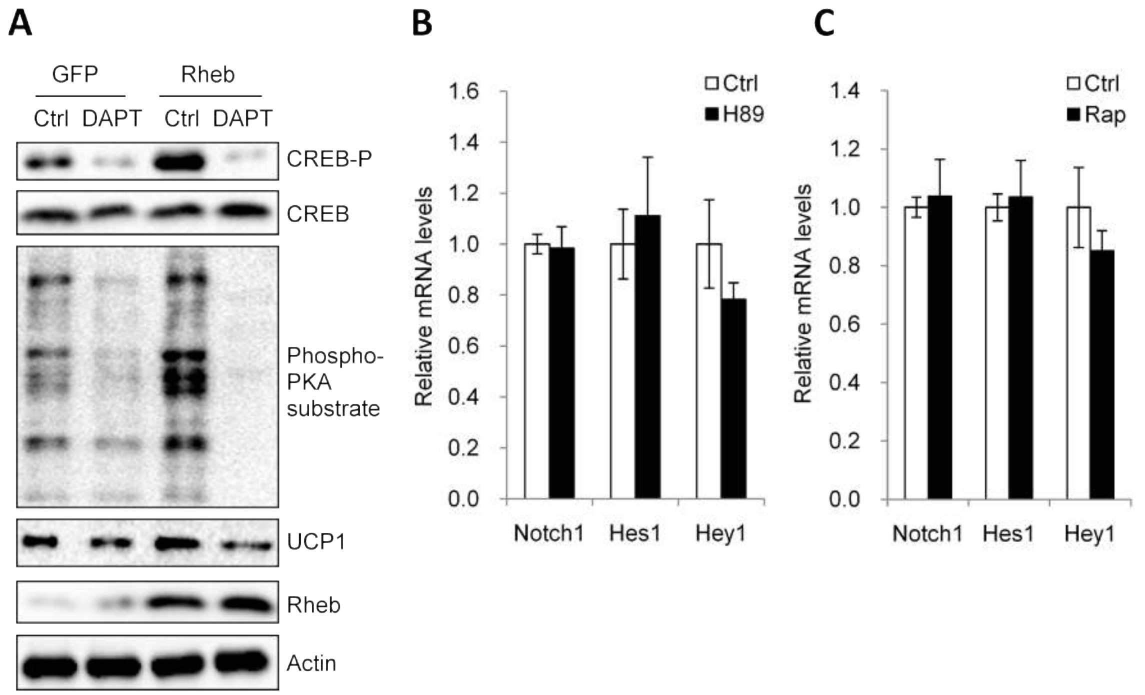

Supplementary Figure S1. Rheb regulates PKA-UCP1 signaling pathway via Notch signaling pathway in brown adipocytes. (A) Primary brown adipocytes were infected with Lentivirus encoding GFP or GFP plus Rheb and then were induced to differentiation. Cells were treated with or without 10 μM DAPT for 24 hrs and analyzed for protein expression using the indicated antibodies. Data were representative of three independent experiments each with a similar result. (B) Primary brown adipocytes were isolated and induced to differentiation. Cells were treated with or without 10 nM H89 for 24 hrs and lysed. The mRNA levels of Notch signaling components were determined by quantitative real-time PCR. Data were mean ± S.E.M. (C) Quantitative real-time PCR analyses of the Notch signaling target genes in primary brown adipocytes treated with or without 20 nM rapamycin for 24 hrs. Data were mean ± S.E.M.

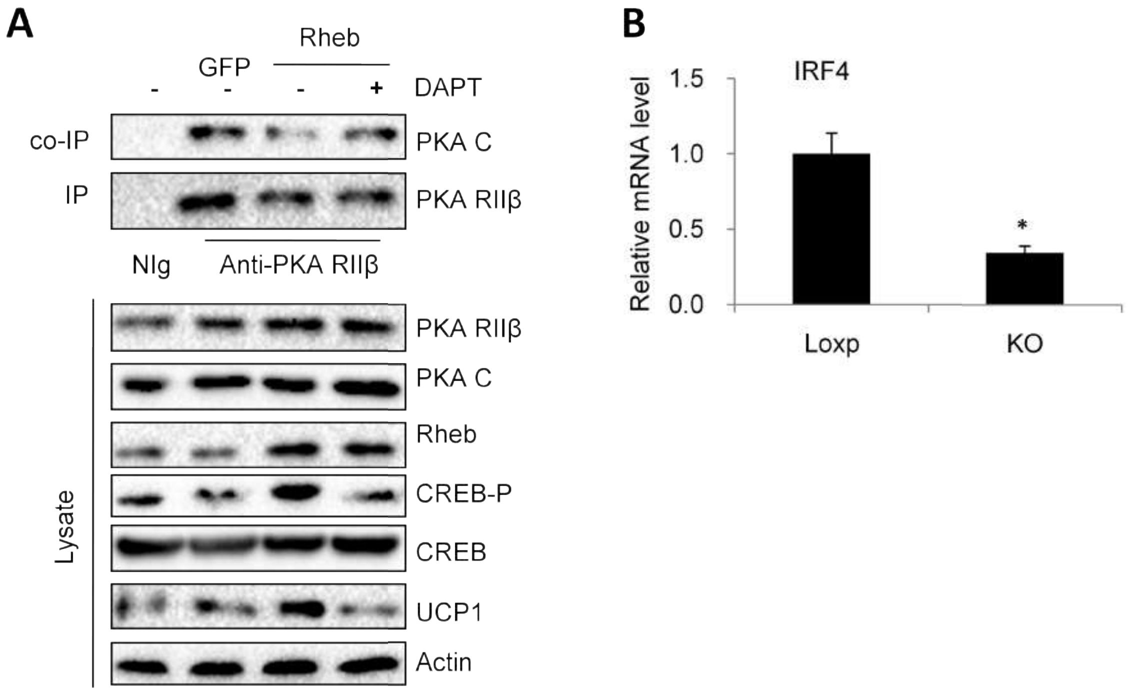

Supplementary Figure S2. Notch signaling activates PKA by suppressing the binding of the regulatory subunit to the catalytic subunit of PKA in brown adipocytes. (A) The immunoprecipitation (IP) of PKA RIIβ and coimmunoprecipitation (co-IP) of PKA C in Rheb-overexpressed primary brown adipocytes which were treated with or without DAPT (10 μM) for 24 hrs. (B) Quantitative real-time PCR analysis of IRF4 mRNA level in BAT from KO and Loxp control mice (n=6/groups). Data were mean ± S.E.M. *P<0.05.

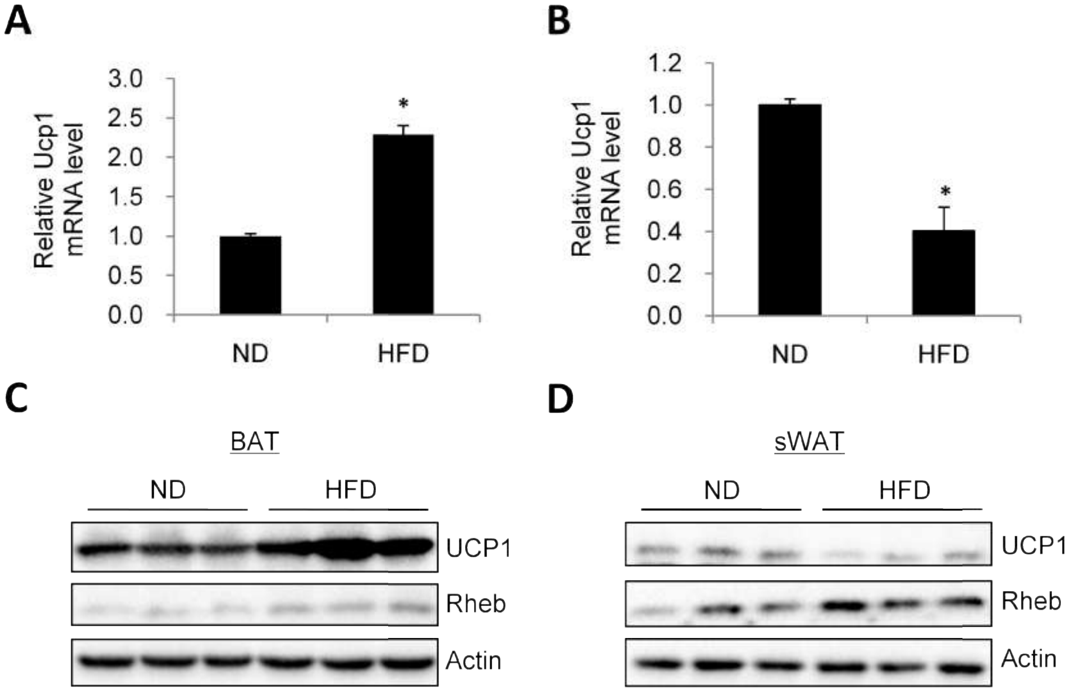

Supplementary Figure S3. HFD induced Ucp1 expression in BAT of mice, but inhibited in sWAT. Quantitative real-time PCR analyses of the UCP1 mRNA level in BAT (A) and sWAT (B) of mice after 8 weeks of HFD (n = 6/group). Data were mean ± S.E.M. *$p < 0.05$. Western blot analyses of the UCP1 and Rheb protein levels in BAT (C) and sWAT (D) of mice after 8 weeks of HFD.

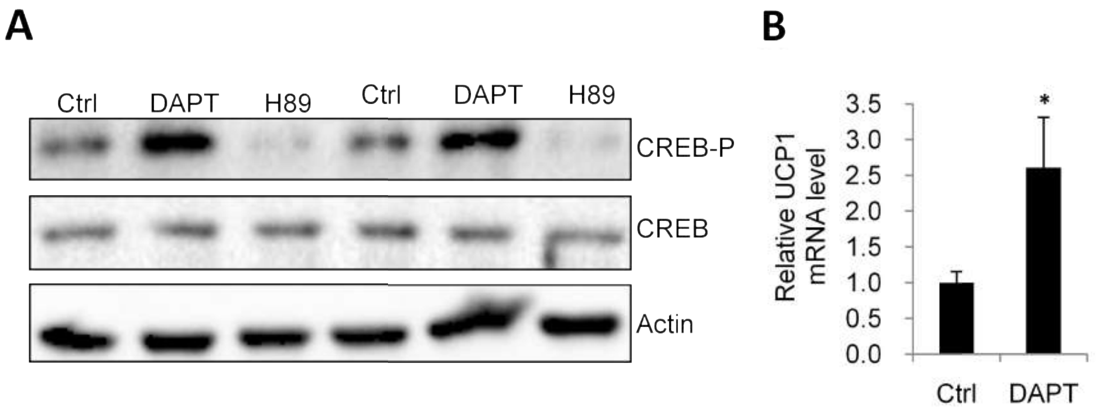

Supplementary Figure S4. Inhibition of Notch signaling promotes browning of white adipose tissue. (A) Western blot analyses of the phosphorylation (P) and protein levels of CREB. Data were representative of three independent experiments each with a similar result. (B) Quantitative real-time PCR analyses of the UCP1 mRNA level in primary white adipocytes which were treated with or without DAPT (10 µM) for 24 hrs. Data were mean ± S.E.M. *$p < 0.05$.

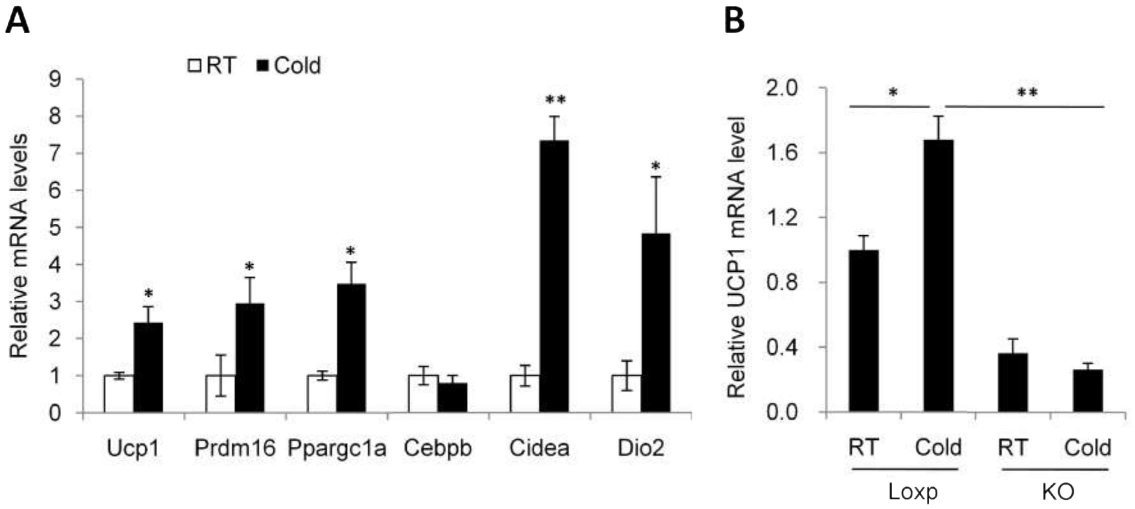

Supplementary Figure S5. Rheb deficiency could suppress cold-induced UCP1 level in BAT of HFD-fed Rheb^{fKO}. (A) Quantitative real-time PCR analyses of thermogenic genes in BAT of cold-exposed mice (n = 3-5/group). (B) Cold exposure–induced UCP1 mRNA expression in BAT of KO and Loxp control mice (n = 3). Data were mean ± S.E.M. *$p < 0.05$; **$p < 0.01$.

II. Supplementary table

Supplementary Table S1. Antibody information.

**Antibody**
S6K-P: Cell Signaling Technology (CST) (catalog number: 9205)
S6K: Cell Signaling Technology (CST) (catalog number: 9202)
Rheb: Cell Signaling Technology (CST) (catalog number: 13879)
Prdm16: Abcam (catalog number: ab106410)
UCP1 : Sigma (catalog number: U6382)
Actin: Sigma (catalog number: A5441)
CREB-P: Cell Signaling Technology (CST) (catalog number: 9198)
CREB: Cell Signaling Technology (CST) (catalog number: 9197)
Anti-activated Notch1 antibody (NICD): Abcam (catalog number:ab8925)
Phospho-PKA Substrate: Cell Signaling Technology (CST)(catalog number:9624)
PKA RIIβ: Abcam (catalog number:ab75993)
Notch1: Cell Signaling Technology (CST) (catalog number: 4380)
HES1: Cell Signaling Technology (CST) (catalog number: 11988)
PKA Ca: Cell Signaling Technology (CST) (catalog number: 4782)
ERK-P: Cell Signaling Technology (CST) (catalog number: 4377)
ERK: Millipore (catalog number: 051152)
